# Supplementary material for: Effects of fasudil on disease spreading in ALS - A MUNIX-based post-hoc analysis of the ROCK-ALS trial
Source: Neurotherapeutics. 2026 Jun 4;23(4):e00936. doi: 10.1016/j.neurot.2026.e00936 (PMC13255067; doi:10.1016/j.neurot.2026.e00936)
Supplement: Multimedia component 1 [file mmc1.docx]

**Effects of Fasudil on disease spreading in ALS - a MUNIX-based post-hoc analysis of the ROCK-ALS trial**

Andreas W. Wolff^*^, Andreas Leha†, Jan C. Koch^‡,§^, Antonia F. Demleitner^*^, Christoph Neuwirth^¶^, Tim Friede^†^, Markus Weber^¶^, and Paul Lingor^*,||,#^

^*^ Clinical Department of Neurology, Klinikum rechts der Isar, School of Medicine, Technical University of Munich, Munich, Germany

^†^ Department of Medical Statistics, University Medical Center Göttingen, Göttingen, Germany

^‡^ Neurozentrum Lindenhof, Sankt Augustin, Germany

^§^ Department of Neurology, University Medical Center Göttingen, Göttingen, Germany

^¶^ Neuromuscular Diseases Unit/ALS Clinic, HOCH Cantonal Hospital St Gallen, St Gallen, Switzerland

^||^ German Center for Neurodegenerative Diseases (DZNE), Munich, Germany

^#^ Munich Cluster of Systems Neurology (SyNergy), Munich, Germany.

Article type: Original Research

Corresponding Author: Paul Lingor, Ismaninger Str. 22, 81675, Munich, Germany; [paul.lingor@tum.de](mailto:paul.lingor@tum.de); Tel: +49-89-4140-8257.


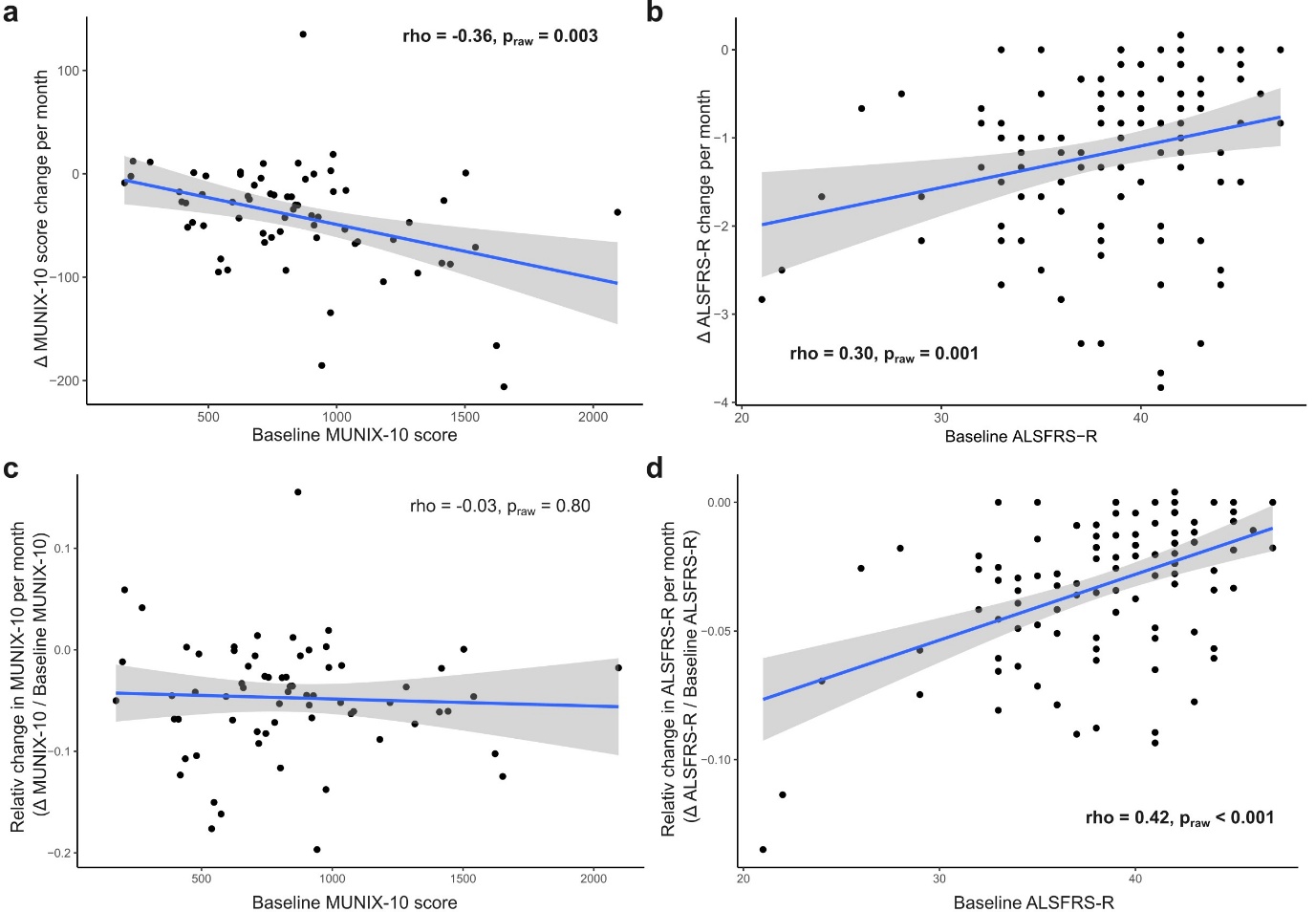


**Supplementary Fig. 1** Correlation between baseline and the monthly change of ALSFRS-R and MUNIX-10 score. a Correlation of MUNIX-10 score change per month and baseline MUNIX-10 score. b Correlation of ALSFRS-R change per month and baseline ALSFRSR. c Correlation of relative monthly change from baseline to baseline in MUNIX-10 score. d Relative monthly change from baseline to baseline in ALSFRS-R.

**Supplementary Table 1:** Spearman correlation coefficients from multiple imputation-based sensitivity analysis

|  | sVC change | ALSFRS-R change |
| --- | --- | --- |
| MUNIX-10 score | rho = 0.04 | rho = **0.30** |
| Log(NfL/MUNIX-10 ratio) | rho = **-0.43** | rho = **-0.48** |
